# Respiratory Illness in a Piggery Associated with the First Identified Outbreak of Swine Influenza in Australia: Assessing the Risk to Human Health and Zoonotic Potential

**DOI:** 10.3390/tropicalmed4020096

**Published:** 2019-06-25

**Authors:** David W. Smith, Ian G. Barr, Richmond Loh, Avram Levy, Simone Tempone, Mark O’Dea, James Watson, Frank Y. K. Wong, Paul V. Effler

**Affiliations:** 1 Department of Microbiology, PathWest Laboratory Medicine WA, Nedlands, WA 6009, Australia; avram.levy@health.wa.gov.au; 2 Faculty of Health and Medical Sciences, University of Western Australia, Nedlands, WA 6009, Australia; paul.effler@health.wa.gov.au; 3 World Health Organization (WHO) Collaborating Centre for Reference and Research on Influenza, at The Peter Doherty Institute for Infection and Immunity, Melbourne, VIC 3000, Australia; Ian.Barr@influenzacentre.org.au; 4 Department of Microbiology and Immunology, University of Melbourne, at the Peter Doherty Institute for Infection and Immunity, Melbourne, VIC 3000, Australia; 5 Sustainability and Biosecurity, Department of Primary Industries and Regional Development, Perth, WA 6151, Australia; richmond.loh@dpird.wa.gov.au; 6 Communicable Disease Control Directorate, Department of Health Western Australia, Perth, WA 6004, Australia; simone.tempone@health.wa.gov.au; 7 School of Veterinary Medicine, Murdoch University, Perth, WA 6150, Australia; M.ODea@murdoch.edu.au; 8 CSIRO Australian Animal Health Laboratory, Geelong, VIC 3219, Australia; James.Watson@csiro.au (J.W.); Frank.Wong@csiro.au (F.Y.K.W.)

**Keywords:** influenza, swine, Australia, human, pandemic

## Abstract

Australia was previously believed to be free of enzootic swine influenza viruses due strict quarantine practices and use of biosecure breeding facilities. The first proven Australian outbreak of swine influenza occurred in Western Australian in 2012, revealing an unrecognized zoonotic risk, and a potential future pandemic threat. A public health investigation was undertaken to determine whether zoonotic infections had occurred and to reduce the risk of further transmission between humans and swine. A program of monitoring, testing, treatment, and vaccination was commenced, and a serosurvey of workers was also undertaken. No acute infections with the swine influenza viruses were detected. Serosurvey results were difficult to interpret due to previous influenza infections and past and current vaccinations. However, several workers had elevated haemagglutination inhibition (HI) antibody levels to the swine influenza viruses that could not be attributed to vaccination or infection with contemporaneous seasonal influenza A viruses. However, we lacked a suitable control population, so this was inconclusive. The experience was valuable in developing better protocols for managing outbreaks at the human–animal interface. Strict adherence to biosecurity practices, and ongoing monitoring of swine and their human contacts is important to mitigate pandemic risk. Strain specific serological assays would greatly assist in identifying zoonotic transmission.

## 1. Introduction

Influenza A viruses (IAV) circulate and evolve continually within bird and swine populations of the world, and are known to be a source of sporadic zoonotic influenza infections, thus presenting a reservoir of potential human pandemic influenza strains. They are known to be present in domestic swine populations internationally, largely as a result of the virus being introduced from humans infected with seasonal influenza viruses [1,2,3]. Some of these human origin viruses persisted and evolved into stable swine lineages through genetic and antigenic drift and virus gene reassortment. They pose an ongoing potential threat to both animal and human health, as demonstrated by the 2009 pandemic, which arose from swine influenza viruses derived from both human and avian IAV that had undergone long term ongoing reassortment [4].

Human infections with swine IAV have been detected since the 1970s and have caused both sporadic cases and outbreaks, such as those reported in the USA [3,5,6]. These zoonotic viruses are mainly of the A(H3N2) subtype with some A(H1N1) and A(H1N2) viruses, which are referred to as variant viruses with a lower-case “v” placed after the subtype, e.g., A(H3N2)v to denote their swine origin.

Prior to 2012 it was believed that Australian domestic swine populations were free of swine lineages of IAV. This was ascribed to negative results from a few limited serosurveys, and the strict quarantine practices preventing importation of pigs and the use of biosecure breeding facilities for domestic swine within Australia. In 2009, a number of outbreaks due to A(H1N1)pdm09 occurred in domestic Australian swine populations through transmission from infected humans, and these have continued to occur sporadically ever since [7]. The virus had little or no apparent ill effect on the health of infected swine and there was no evidence that this resulted from, or led to, enzootic IAV in Australian swine, and so was not seen to be a threat to human or animal health nor a potential source of pandemic viruses.

However, in 2012 an outbreak of respiratory illness and death occurred amongst pigs in a biosecure piggery near Perth, the capital of Western Australia [8], shown to be due to several previously unidentified swine IAV containing genes of human origin [9]. A mild respiratory illness was also reported by several of the workers. A combined investigation of this outbreak by the Department of Agriculture and Food, Western Australia (DAFWA) and the Communicable Disease Control Directorate (CDCD) of the Department of Health Western Australia led to the detection of a number of genetically distinct divergent IAV in the swine.

An extensive phylogenetic analysis indicated that the IAV in the Western Australian swine contain human origin genes not found in human populations for several decades, and that they are distinct from those identified in swine in the rest of the world, including those subsequently identified in swine in a piggery in Queensland [9], approximately 4000 km distant. This ongoing circulation and evolution of IAV in the Western Australian swine population for up to several decades revealed an unrecognized potential zoonotic threat [9].

In view of the known risk of transmission of IAV from pigs to humans in other countries [2,6], interventions were undertaken to investigate and mitigate the risk of human infection. This paper describes the investigations undertaken into this outbreak to assess the potential risk to human contacts and to determine whether zoonotic transmission had occurred. The importance of these investigations has been further emphasized by the recent description of the first human infection with a variant swine H3N2, acquired within Australia [10]. This occurred outside Western Australia following exposure to exhibition swine at an agricultural fair, and was confirmed to be due to a virus similar to, but distinct from, the swine viruses found in Western Australian and Queensland swine.

## 2. Materials and Methods 

Active surveillance for respiratory illness among the workers at the affected piggery was commenced when the outbreak was notified to the CDCD. Those who developed any respiratory illness were assessed by a medical practitioner, and mid-turbinate nasal and throat swabs were collected using flocked swabs (FLOQSwabs, Copan Diagnostic Inc, Murietta, CA, USA). Swab samples were placed in virus transport medium and transported to PathWest Laboratory Medicine WA (PathWest, Perth, Australia) at 4 °C. Serum samples from symptomatic individuals were collected by standard venipuncture, then stored and transported at 4 °C, with a convalescent sample collected 10–14 days later. Blood samples for the serosurvey were collected in the same manner.

PCR tests at PathWest used an in-house duplex assay which included specific real-time RT-PCRs, respectively, directed at the influenza A matrix gene, the seasonal A(H3N2) HA gene, the seasonal A(H1N1)pdm09 HA gene and the influenza B matrix gene [11]. PCR testing for other respiratory viruses in the human contacts was carried as previously described [12] and swabs were also inoculated onto an MDCK cell monolayer for virus isolation. Rhinovirus speciation was based on 5’NTR sequence [12] and the characterization of matrix, HA and NA genes of influenza A viruses was carried out by conventional Sanger sequencing.

Testing for antibodies to influenza A and B was performed by complement fixation titer (CFT) at PathWest, while haemagglutination inhibition (HI) antibody test for the human sera and the antigenic characterization of the swine IAV isolates was carried out at the World Health Organization Collaborating Centre for Reference and Research on Influenza (WHOCC), Melbourne, Victoria. Comparison of titers used a Kruskal–Wallis test performed on the log_2_ converted values.

## 3. Results

**Figure 1:**
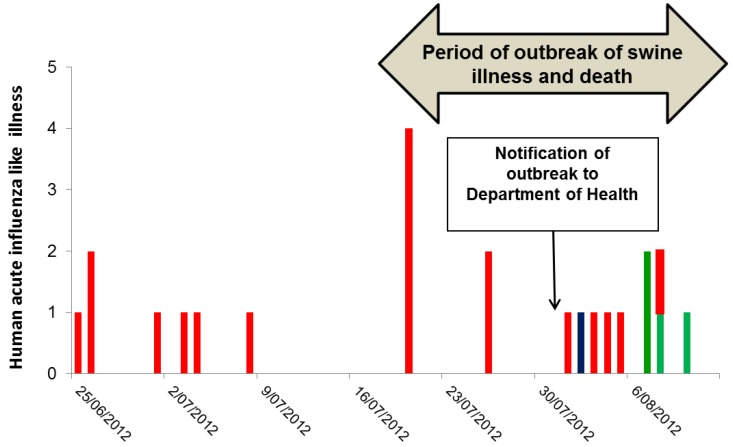
Date of onset of influenza-like illness and respiratory virus detections in 23 workers at a piggery in WA preceding and during an outbreak of respiratory tract illness and deaths in swine. The red columns are those with respiratory illness without laboratory confirmation either because they were not tested (17 workers) or because the results were negative or inconclusive. The blue column is confirmed seasonal A(H3N2) and the green are confirmed RV infections. Initial serologic testing at the DAFWA had suggested influenza infection and on 4 August 2012, a site visit was made by officers from the CDCD and the DAFWA.

The outbreak of serious illness among swine began in mid-July 2012, and then gradually subsided over the course of a few weeks (Figure 1) [8].

No symptomatic humans were present during the initial site visit. However, 20 upper respiratory tract swabs were collected from symptomatic swine for testing by PCR and culture. 

Initial PCR testing of the swine samples at PathWest were positive for influenza A matrix gene, but negative for the HA genes of the contemporaneously circulating A(H1N1)pdm09 and A(H3N2) viruses, indicating a possible novel HA type. This was later confirmed by the WHOCC in Melbourne and the Australian Animal Health Laboratories in Geelong, Victoria [9].

Following the initial testing, which indicated that these were likely to be nonseasonal IAV, suggesting a possible swine virus or an exotic human virus, the piggery was placed in quarantine [8] and surveillance for respiratory illness in staff at the piggery was commenced. A protocol was put in place to ensure that staff developing a respiratory illness were assessed and tested urgently by PCR, treatment with oral oseltamivir at 75mg twice daily was immediately provided, and the person was isolated at home until cleared of infection. Acute and convalescent serum samples were collected from these symptomatic workers and arrangements were made for collection of convalescent samples 10–14 days later. The use of personal protective equipment (PPE) by the staff in contact with swine was also emphasized, to reduce respiratory virus transmission.

Phylogenetic analysis of the swine IAVs characterized them as novel reassortant viruses containing only gene segments of human origin: two H1N2 reassortant viruses (H1N2/A/sw/WA/2577899R/2012 and H1N2/A/sw/WA/2577896X/2012) and one H3N2 reassortant virus (H3N2/A/Swine/WA/2577766G/2012) [9]. One of the A(H1N2) viruses (2577899R) and the A(H3N2) virus contained only segments that had not been detected in human populations for up to decades. These were chosen for the comparative serology. The other A(H1N2) virus (2577896X) had the same HA and NA genes as H1N2/2577899R, but the internal genes of the recent A(H1N1)pdm09 virus. The absence of any known matching viruses recently circulating in human populations internationally, and the ongoing circulation and evolution of these viruses within swine populations confirmed them as enzootic swine influenza viruses. 

On 10 August, a week after the notification of the outbreak and following confirmation of the virus identification, seasonal influenza vaccination was carried out for the piggery workers, using the 2012 southern hemisphere trivalent influenza vaccine, and prophylaxis with oral oseltamivir 75 mg bd was provided for two weeks while vaccine responses were developing. This was done to reduce the risk of workers being infected with the variant virus, to reduce possible confounding seasonal influenza infections in the workers, to reduce the risk of further introduction of circulating human influenza viruses into the swine population, and to minimize the risk of mixed infections and reassortment between human and pig viruses [13].

Further testing of samples from 131 swine of various ages was carried out at PathWest in order to define the extent of the risk to human contacts. Of these, 43 (32.8%) had IAV detected based on a positive PCR for the matrix gene. Sanger sequencing of the products of the HA and NA genes indicated that six swine had a novel H1N2 virus, three (6.9%) had a novel H3N2 virus, one (2.3%) had A(H1N1)pdm09 infection, and two (4.6%) had likely seasonal A(H3N2) virus. The remainder could not be characterized by this sequencing. These results were consistent with whole genome sequencing result of the reassortant A(H1N2) and A(H3N2) swine viruses [9]. Sanger sequencing of the matrix gene of the six novel H1N2 viruses all matched with seasonal A(H1N1)pdm09 virus sequences, indicating that they were highly likely to be the H1N2/A/sw/WA/2577896X/2012 reassortant.

On 24 August 2012, three weeks after the notification of the outbreak to CDCD and two weeks after the vaccination program, 69 of 70 workers at the piggery completed a survey enquiring about symptoms of influenza-like illness during the period beginning one month prior to the outbreak in swine. Overall 27/69 (39%) of workers reported a mild respiratory illness in the month before or during the swine outbreak, and 23 of these 27 ill workers could recall their onset date. Seven had onset prior to the outbreak, seven during the swine outbreak but prior to notification, and nine after notification of the outbreak (Figure 1). Of those nine cases, eight were swabbed. One yielded a seasonal A(H3N2) virus, and three had a rhinovirus (RV), comprising two RV-C and one RV-A. The remaining four were negative for respiratory viruses. 

Paired acute and convalescent serum samples were available for five cases (Table 1), including the individual with PCR-confirmed H3N2 infection who demonstrated an increase in HI antibodies to seasonal A(H3N2) from <1:10 to 1:320 between acute and convalescent sera, compared with an increase from 1:20 to 1:80 to the swine A(H3N2). Two others showed ≥ 4-fold rises in HI titers to A(H1N1)pdm09, one of whom also had a 4-fold rise in antibody titer to seasonal A(H3N2). However, both had been vaccinated between the acute and convalescent sample and this may have accounted for these results. The remaining workers did not show any significant HI changes.

Serum samples were requested at the time of the survey and 57/69 workers, 48 of whom had received the seasonal influenza vaccine two weeks earlier, agreed to provide serum samples. HI titers to swine A(H1N2) and A(H3N2) and to the vaccine A(H1N1) and A(H3N2) were determined (Table S1).

All of the nine unvaccinated workers had HI titers ≤1:80 to the swine A(H1N2) and all of these were lower than or equivalent to the titers to the vaccine A(H1N1). Seven had HI titers <40 to the swine A(H3N2), one had an HI titer of 40 to the swine A(H3N2) but this was lower than the titer to the vaccine A(H3N2). However, the remaining worker had a significantly higher HI titer to the swine A(H3N2) virus (1:320) than to the seasonal A(H3N2) virus (1:80). In summary, we found one unvaccinated worker who had serological evidence of a possible infection with the swine A(H3N2) virus. For the others, we either found no significant titers to the swine viruses or we could not exclude cross-reacting antibody due to infection with seasonal viruses, as represented by the vaccine strains. 

The other 48 workers who underwent testing had received the seasonal influenza vaccine as part of the outbreak response. Therefore, we anticipated that we would detect vaccine-induced antibodies that might mask responses to infection with the swine viruses. Therefore we examined the likely effect of vaccination on antibody levels to the swine viruses by comparing HI titers in 48 vaccinated workers with those in the nine unvaccinated workers. Forty-six of the samples from vaccinated workers had sufficient volume to complete the testing for all viruses, while two further workers had sufficient sample to test for antibodies to the swine A(H1N2) and the vaccine A(H1N1) viruses, but not for antibodies to the A(H3N2) viruses (Table 2). 

Vaccination had no effect on the HI titers to the swine A(H1N2) virus or the vaccine A(H1N1) strain, so that the antibody titers (Table S1) can be interpreted independent of vaccination. For the combined vaccinated and unvaccinated workers, there were a total of 24 workers who had HI titers ≥40 to the swine A(H1N2) virus, but 23 of these workers had equivalent or higher titers to the vaccine A(H1N1) virus, so that cross-reacting antibody from seasonal influenza A infection or vaccination could not be excluded. However, the remaining worker had a 16-fold higher titer to the swine A(H1N2) virus, suggesting possible infection with that virus. 

In contrast, vaccination resulted in significant and similar increases in antibody titers to both the swine and seasonal A(H3N2) viruses. The results for the unvaccinated workers (see above) identified one worker with a possible swine A(H3N2) infection. Among the vaccinated workers, 35/46 had HI titers ≥40 to the swine A(H3N2) virus, of which 17 had higher titers to the swine virus than to the vaccine virus. Eight of these were only two-fold higher and were discounted as this difference was not significant. However, there were nine samples where the HI titer was between four-fold and 32-fold higher to the swine virus, which cannot be attributed to vaccination or seasonal virus infection. 

These data suggest that, at least in some of the workers, there is evidence that they may have had an infection with one of the swine influenza viruses as their antibody titers could not be explained by cross-reacting antibody from their recent vaccination or seasonal influenza virus infection. However, we cannot exclude higher antibody titers due to past infection with human origin viruses that were serologically closely-related to the swine viruses, as we do not have a demographically matched control group that were not exposed to the swine influenza viruses. 

## 4. Discussion

Influenza A viruses circulate and evolve continually within swine populations and represent a reservoir of potential human pandemic influenza strains. These viruses occasionally cross into human populations following contact with swine, including exhibition swine at agricultural events [6,14,15]. Until 2012 it was thought that Australia was free of this risk, as enzootic influenza had not been detected in local swine populations previously. Characterization of the outbreak described here clearly demonstrated that human influenza viruses have been regularly entering Australian swine populations from humans, probably for decades, and that circulation and reassortment has persisted within swine [9]. Infection of humans with variant influenza viruses arising from piggeries has not yet been documented, but there is a continuing possibility that swine viruses with pandemic potential may spread to humans in contact with swine within Australia. That appears to have happened with the recent first variant influenza virus infection of a human in Australia, associated with exposure to exhibition swine [10].

The Western Australian piggery outbreak was the first to identify enzootic influenza virus infection in swine in Australia [9], and we have described the approach taken to determine whether swine-to-human transmission had occurred and to mitigate the risk of further transmission between swine and humans. That included reinforcing personal hygiene measures and providing seasonal influenza vaccination for previously unvaccinated workers [16]. Active surveillance for respiratory illness in the workers was instituted as soon as the outbreak was notified, but none of the subsequent respiratory illnesses occurring could be attributed to the swine influenza viruses by PCR testing or by serology. 

The limitations of using virus detection for assessing infection of human contacts are known, especially where long term exposure is likely and the illness is mild, so that the likelihood of identifying and sampling acute infections is low [17]. Serosurveys have the advantage of detecting past as well as current infections, but are problematic for influenza diagnosis due to the multiple exposures and vaccinations that occur during life and the cross-reactivity of influenza antibodies across different strains of the virus. Adults have diverse immune backgrounds to influenza and therefore have complex serological responses to infections with new influenza viruses. In our study, the interpretation of the results was further complicated by the vaccination program that had been undertaken two weeks prior to the serosurvey. This may have masked antibody responses to the swine viruses, which are difficult to separate from possible cross-reactions from the vaccine response and/or past natural exposure to human viruses. 

However, we did not find any evidence that vaccination with the seasonal influenza vaccine influenced the antibody titer to the swine A(H1N2) virus. So the high titer HI antibody to this virus with a low titer to the seasonal A(H1N1)pdm09 virus that we found in one worker could not be explained by vaccination, and indicated possible infection with the swine virus. With the swine A(H3N2) viruses, we did find that vaccination increased titers to both the swine A(H3N2) and the vaccine A(H3N2) viruses, but several workers had HI titers to the swine A(H3N2) virus that were at least fourfold higher than the responses to the vaccine A(H3N2) virus. That result is not consistent with a vaccine response, and again suggests possible infection with a swine A(H3N2) virus. However, the HI titers to the swine A(H1N2) and A(H3N2) viruses could possibly have been due to past natural infection with antigenically similar viruses that circulated within human populations. 

The challenges associated with using serosurveys to assess zoonotic influenza infections have been recently reviewed [17]. The authors recommended that, in order to identify antibodies specific to the animal viruses, the protocol should include testing for antibodies to both human and variant viruses for cross-reactivity; the use of two different serological assays, one of which should be a neutralization assay, to improve specificity; and having matched control samples from persons not in contact with the animals. Many of the piggery workers were from overseas, which meant that they had potentially been exposed to a different spectrum of influenza A viruses that may have resulted in antibodies that cross-reacted with the swine influenza viruses. Unfortunately, we could not access satisfactory control sera to exclude that possibility. In view of that limitation, we elected not to proceed with neutralization assays as it was unlikely to provide definitive results. 

The ongoing circulation of influenza viruses within swine populations in Australia, with evidence of transmission between humans and swine overseas, and the likely recent human infection acquired within Australia has made us aware of potential for the generation of zoonotic infections. This also raises the possibility, albeit low, of human pandemic strains arising within Australia following zoonotic infection with a swine IAV. While commercial piggeries have not yet been shown to be a source of zoonotic infection with swine IAV, precautions are recommended to mitigate this risk [16]. Further studies are needed to identify the extent and diversity of influenza viruses in swine and other animals in regular contact with humans, and to conduct studies to determine the frequency of transmission to those human contacts. 

The animal health sector has recently provided updated recommendations for maintaining biosecurity at piggeries, and for the management of outbreaks [18,19]. In addition it is important to restrict casual contact with swine at agricultural events, to carry out seasonal vaccination programs for piggery workers and others with regular swine contact, to maintain surveillance of people working at the human–animal interface and to have early structured investigation of outbreaks of respiratory illness in the animals or their human contacts [13,16].

## Figures and Tables

**Table 1:** Acute and convalescent serum HI titers to swine and vaccine influenza A viruses for five workers with respiratory illness who provided acute and convalescent samples. Significant titer changes are in bolded text.

	Vaccine Given	Respiratory Illness between Samples	Sample	Swine Viruses		Vaccine Viruses	
				H1N2	H3N2	H1N1pdm09	H3N2
				A/WA/896X/2012	A/WA/766G/2012	A/California/7/2009	A/Perth/16/2009
1	Yes	No	Acute	<10	10	**<10**	**<10**
			Convalescent	<10	<10	**320**	**40**
2	Yes	Yes ^1^	Acute	<10	**20**	**10**	**<10**
			Convalescent	10	**80**	**40**	**320**
3	No	No	Acute	40	<10	20	20
			Convalescent	40	<10	40	10
4	Yes	No	Acute	20	20	40	80
			Convalescent	20	20	40	160
5	Yes	No	Acute	**10**	80	**40**	80
			Convalescent	**40**	160	**320**	160

^1^ PCR-proven seasonal A(H3N2) infection.

**Table 2:** A comparison of the mean HI values in vaccinated and unvaccinated workers using a Kruskal–Wallis test on the log_2_-transformed HI titer, where HI titers <10 were assigned a notional value of 1. The one worker with a confirmed acute seasonal A(H3N2) infection was excluded.

Virus	Median of the log_2_-Converted HI Titers		*p*-Value
	Unvaccinated	Vaccinated ^a^	
WA Swine H1N2 ^1^	0.00	3.32	0.76
Human vaccine A/H1N1 09pdm ^2^	6.32	6.32	0.87
WA Swine H3N2 ^3^	3.32	6.32	0.0004
Human vaccine A/H3N2 ^4^	5.32	6.32	0.025

^1^ A/Swine/WA/2577896X/2012 H1N2, ^2^ A/California/7/2009 H1N1pdm09, ^3^ A/Swine/WA/2577766G/2012 H3N2, ^4^ A/Perth/16/2009 H3N2. ^a^ Only 46 workers were able to be tested for antibodies to the A(H3N2) viruses due to insufficient sample volume remaining for the final two workers.

## Supplementary Materials

The following are available online at https://www.mdpi.com/2414-6366/4/2/96/s1, Table S1: HI values in vaccinated and unvaccinated workers. The one worker with a confirmed acute seasonal A(H1N2) infection was excluded. Highlighted results are those where the titer to a swine IAV was ≥40 and at least four-fold higher than the titer to the vaccine strain.
