# Supplementary material for: Respiratory Illness in a Piggery Associated with the First Identified Outbreak of Swine Influenza in Australia: Assessing the Risk to Human Health and Zoonotic Potential
Source: Trop Med Infect Dis. 2019 Jun 25;4(2):96. doi: 10.3390/tropicalmed4020096 (PMC6632059; doi:10.3390/tropicalmed4020096)
Supplement: Supplementary file 1 [file tropicalmed-04-00096-s001.pdf]

Supplementary Table 1: HI values in vaccinated and unvaccinated workers. The one worker with a confirmed acute seasonal A(H1N2) infection was excluded. Highlighted results are those where the titre to a swine IAV was ≥40 and at least four-fold higher than the titre to the vaccine strain.

Vaccinated workers				
ID	A/ H1N2/ WA/ 896X/ 2012	A/ H1N1pdm09/ Calif/ 7/ 2009	A/ H3N2/ WA/ 766G/ 2012	A/ H3N2/ Perth/ 16/ 2009
1	10	80	160	40
2	160	320	1280	640
3	80	160	40	160
4	80	160	40	1280
5	<10	20	<10	<10
6	<10	320	<10	40
7	10	40	40	40
8	<10	<10	80	<10
10	<10	160	<10	640
11	<10	320	40	80
13	<10	80	160	80
14	<10	80	640	80
15	<10	80	10	40
16	160	160	80	80
17	<10	80	160	160
18	40	80	80	40
19	<10	320	320	160
20	<10	20	80	80
22	10	40	80	320
23	40	80	320	80
24	40	40	<10	10
25	40	320	640	1280
27	160	1280	1280	160
29	40	80	80	40
30	<10	40	<10	80
31	80	160	40	160
32	<10	40	160	<10
33	80	160	40	<10
34	80	160	80	160
35	<10	40	80	<10
37	160	320	40	80
38	<10	160	20	80
39	<10	160	160	320
40	<10	40	<10	<10
41	20	40	20	160
42	40	320	160	10
43	<10	80	80	160
44	<10	640	320	320
45	80	640	40	80
47	10	40	20	160
48	<10	160	320	640
49	40	40	20	10
50	<10	320	160	160
51	160	320	80	640
52	20	640	320	640
53	<10	40	80	40
55	80	<10	ND	ND
56	160	160	ND	ND

Unvaccinated workers				
ID	A/ H1N2/ WA/ 896X/ 2012	A/ H1N1pdm09/ Calif/ 7/ 2009	A/ H3N2/ WA/ 766G/ 2012	A/ H3N2/ Perth/ 16/ 2009
28	<10	<10	<10	<10
58	<10	10	20	<10
36	80	80	10	<10
12	<10	40	<10	40
9	40	640	320	80
26	<10	80	<10	80
21	80	320	10	80
45	80	640	40	80
54	<10	<10	<10	<10
